# Resveratrol improves the prognosis of rats after spinal cord injury by inhibiting mitogen-activated protein kinases signaling pathway

**DOI:** 10.1038/s41598-023-46541-x

**Published:** 2023-11-13

**Authors:** Shunli Kan, Chengjiang Liu, Xinyan Zhao, Sa Feng, Haoqiang Zhu, Boyuan Ma, Mengmeng Zhou, Xuanhao Fu, Wei Hu, Rusen Zhu

**Affiliations:** Department of Spine Surgery, Tianjin Union Medical Center, 190 Jieyuan Road, Hongqiao District, Tianjin, 300121 China

**Keywords:** Cell death in the nervous system, Cellular neuroscience, Molecular neuroscience

## Abstract

Spinal cord injury (SCI) is a serious condition that results in irreparable nerve damage and severe loss of motor or sensory function. Resveratrol (3,4′,5-trihy- droxystilbene) is a naturally occurring plant-based polyphenol that has demonstrated powerful antioxidative, anti-inflammatory, and anti-carcinogenic pharmaceutical properties in previous studies. In the central nervous system, it promotes neuronal recovery and protects residual function. However, the role of resveratrol in SCI recovery remains elusive. In this study, the potential mechanisms by which resveratrol affect SCI in rats were assessed by constructing a contusion model of SCI. Resveratrol was intraperitoneally administered to rats. Behavioral scores and electrophysiological examinations were performed to assess functional recovery. After magnetic resonance imaging and staining with hematoxylin and eosin (HE) and Luxor Fast Blue (LFB), tissue recovery was analyzed. Immunofluorescence with NeuN and glial fibrillary acidic protein (GFAP) was employed to evaluate neuronal survival and glial changes. TdT-mediated dUTP nick end labeling (TUNEL) assay was performed to examine apoptotic rates. Moreover, network pharmacology was performed to identify relevant pathways of resveratrol for the treatment of SCI. Lastly, ELISA was performed to detect the expression levels of interleukin-1β (IL-1β), tumor necrosis factor-α (TNF-α), and IL-6. Our findings revealed that resveratrol dramatically improved the hindlimb locomotor function and their electrophysiological outcomes. Notably, lesion size was significantly reduced on magnetic resonance imaging. HE and LFB staining exposed increased sparseness of tissue and myelin. GFAP and NeuN immunofluorescence assays at the lesion site determined that resveratrol boosted neuronal survival and attenuated glial cell overgrowth. In addition, resveratrol reduced the density and number of TUNEL-positive cells in rats after injury. Additionally, gene ontology analysis revealed that the enriched differentially expressed protein was associated with the JNK/p38MAPK (c-jun N-terminal kinase/p38 mitogen-activated protein kinase) signaling pathway. Following resveratrol treatment, the expression levels of IL-1β, TNF-α, and IL-6 were decreased. In summary, the administration of resveratrol protects motor function and neuronal survival in rats after SCI. Furthermore, resveratrol exerts an anti-inflammatory effect by blocking the JNK/p38MAPK signaling pathway.

## Introduction

Spinal cord injury (SCI) is a severe condition that causes irreparable nerve damage and severe loss of motor or sensory function in the limbs below the injury site, with a high mortality rate for injured individuals^1^. Patients with SCI experience massive economic and psychological costs and compromised quality of life. SCI comprises both primary and secondary injury components. The latter is predominantly caused by hemorrhage, ischemia, cell death, and disruption of the blood-spinal cord barrier at the site of the injury following mechanical injury^2^. Secondary damage on top of this further exacerbates the situation, leading to spinal cord inflammation, apoptosis, lipid peroxidation, and necrosis^3^. The inflammatory response in this process is closely linked to the secondary damage and can influence or even determine the recovery and prognosis of neurological function. Inflammation is known to aggravate tissue necrosis^4^. Therefore, overcoming inflammatory response at the early stage is a valuable treatment strategy for SCI.

Resveratrol (3,4′,5-trihy- droxystilbene) is a naturally occurring plant-based polyphenol that has demonstrated powerful antioxidative, anti-inflammatory, and anti-carcinogenic pharmaceutical characteristics in previous studies^5^. It has been prescribed for the treatment of cerebral apoplexy and exhibited positive clinical outcomes^6^. Importantly, this phytocide is well absorbed and rapidly and extensively metabolized in the body. In recent years, resveratrol has been extensively studied for its anti-tumorigenic effects, weight loss benefits, and anti-depressant properties^7^. Several studies have documented that resveratrol can scavenge free radicals and is effective in inhibiting lipid peroxidation and governing the activity of oxidation-related enzymes^8,9^. Besides, its antioxidant properties are well established. Earlier studies have established its unique protective effect on the central nervous system. On the one hand, it improves the prognosis of ischemia–reperfusion injury in the brain. On the other hand, it enhances cognitive and memory function to varying degrees following brain injury^10^. At the same time, resveratrol inhibits inflammation and stimulates neuronal recovery after central nervous system injury. In addition, resveratrol confers a neuroprotective impact by regulating apoptosis and autophagy after neurological injury^11^. However, the mechanism by which resveratrol influences the inflammatory response to SCI, as well as its detailed molecular mechanisms, remain to be elucidated. Our investigation sought to identify and experimentally validate its molecular targets for the treatment of spinal cord injury after demonstrating that resveratrol improves recovery in rats following spinal cord injury using a rat model.

The network pharmacology approach has been extensively employed to determine drug-active ingredients and their molecular targets. Protein kinases that respond to mitogens are referred to as mitogen-activated protein kinases (MAPKs) and belong to the highly conserved serine/threonine protein family^12^. It regulates various cellular processes, including cell growth, proliferation, differentiation, and death^13^. In the present study, a rat model of SCI was established to explore the molecular mechanisms of resveratrol on functional recovery after SCI injury. Through network pharmacology studies, the MAPK pathway was identified as a practical mechanism by which resveratrol inhibits inflammation at the site of injury after SCI in rats.

## Material and methods

### Animals

A total of 44 female (200–240 g) Wistar rats were purchased from the Laboratory Animal Centre of the Academy of Military Medical Sciences. Humidity and temperature were controlled in a 12-h diurnal cycle. The rats were divided into three groups: injury group (n = 16), injury and saline; Sham group (n = 12), only laminectomy without spinal cord injury; Resveratrol group (n = 16), injury and 10 mg/kg resveratrol (R8350, Solarbio Company). Intraperitoneal injection of resveratrol was initiated immediately following surgery and continued daily until the 7th day. The experiments were approved by the Ethics Committee of Tianjin Union Medical Center (IRM-DWLL-2019039). 10 μm.

### Establishment of the SCI rat model

Modeling spinal cord contusion injuries was implemented using the MASCIS Impactor Model III. Rats were anesthetized using isoflurane (2% in O_2_ delivered at 2 L/min), and the spinal cord was exposed after T10 laminectomy. A 10 g nodule was dropped from a height of 25 mm and impacted the spinal cord, causing a spinal cord contusion. After the surgery, all animals were placed into a heated cage to prevent core temperature loss. After modeling, the rats were made to urinate artificially twice daily, until their autonomous urination function was restored. Laminectomy was the only procedure performed in the sham group. All experiments were carried out in accordance with relevant guidelines and regulations and reported following the ARRIVE guidelines.

### BBB locomotor score

In this experiment, the BBB locomotor score was used to assess neural function at 0, 7, 14, 21, 28, 35, 42, and 56 days after SCI. It utilizes a scale of 0–21 points with 22 grades. The test was executed on the same time of day for all the groups and performed weekly for up to 8 weeks post-SCI. The scoring system is based on the observation of the hind limb movement in the open space over a period of five minutes. Each rat was assessed by two experienced observers blinded to the group assignments.

### Catwalk gait analysis system

CatWalk (Noldus, Wageningen) analysis was used to investigate dynamic walking patterns. The core components of the system comprise an illuminated glass plate and a camera under the walkway. Rats were trained to cross the vitreous walkway, and each footprint was analyzed into pixels of digital brightness footprint ranging from 0 to 255 (arbitrary units). The analysis system CatWalk XT (version 10.6) was employed to quantify the data and generate digital parameters, including qualitative and quantitative assessments of individual footprints and gait parameters to evaluate the overall hind paw recovery, maximum contact area, regularity index, swing, and stride length. Prior to recording, all rats were placed on CatWalk glass plates for 20 min to avoid stress-related biases.

### Magnetic resonance imaging

MR images were used to show the morphological changes after SCI, and a 3.0 T spectrometer (Discovery MR750, GE Healthcare, USA) was used to acquire the MR images. Parameters include slice thickness and slice gap of 2 and 0.5 mm, respectively. To quantify the lesion regions shown on MR images, the percentage of lesion area to the area of T9-T11 was calculated. Using ImageJ software (National Institutes of Health), T2 intensity was calculated from T2-weighted images of the lesion sites.

### Electrophysiological evaluation

The rats were anesthetized 8 weeks after injury. Electrophysiological devices were then utilized to record somatosensory evoked potential (SEP). A motor-evoked potential (MEP) was also used to monitor motor tracts within injured spinal cords. The experiment examined evoked potentials in the right hind limb of rats. A constant stimulator generates a 5.1 Hz square wave for 0.1 ms to stimulate the posterior tibial nerve. Regarding SEP, the current intensity was 2 mA. A constant current stimulus was applied to the motor cortex to generate MEP. Three different electrodes were placed on the corresponding parts of the rat. After checking the circuit connections, MEP was recorded at least 5 times.

### Histological evaluation

#### Luxol fast blue (LFB) staining

Anesthetized rats were transcranially perfused with 0.9% NaCl followed by 4% paraformaldehyde at week 4 post-SCI. From the focal point of the lesion, approximately 10 mm of spinal cord tissue was dissected. After 24 h at 4 °C, the samples were immersed in 4% paraformaldehyde. Lastly, the sections were stained with LFB. Image J was used to quantify microscopy images.

#### Hematoxylin–Eosin (HE) staining

Spinal cord tissue was fixed in 4% paraformaldehyde for 24 h and then sequentially immersed in a series of alcohol gradients for systematic dehydration. After the tissues were immersed in paraffin wax, the blocks were sectioned into 10 μm-thick sections. Xylene was used for dewaxing, followed by sequential rehydration with decreasing concentrations of ethanol. HE staining solution was used for the staining process. Afterward, a light microscope was used to capture images of the slices after staining. Image J was used for quantitative analysis of microscopy images.

#### Immunofluorescence staining

Immunofluorescence staining was performed on spinal cord sections following dewaxing with xylene, treatment with various concentrations of ethanol, and triple washing with PBS. After incubation with H_2_O_2_, the sections were incubated with primary antibodies at 37 °C for 60 min and then overnight in the refrigerator. The primary antibodies used in the experiments were Goat Anti-Rat GFAP Antibody (1:400; Abcam) and Mouse Anti-NeuN Antibody (1:200; Abcam). Then, after washing three times with PBS, the sections were incubated with the secondary antibody at room temperature for 60 min. Thereafter, they were washed with PBS, followed by counterstaining with DAPI. Finally, the immunofluorescence images were visualized using Olympus Fluoviewer (TH4-200) and further quantified by ImageJ software (National Institutes of Health, USA).

#### TdT-mediated dUTP nick end labeling (TUNEL) staining

In Situ Cell Death Assay Kit (Roche, Mannheim) was utilized to perform TUNEL staining. Briefly, the sections were rehydrated as described in the immunofluorescence staining procedure. After 30 min of incubation at room temperature and two washes with PBS, proteinase K solution was subsequently added. Next, the samples were incubated for 30 min at room temperature and then washed twice with PBS. Each sample was incubated for an additional 10 min with diaminobenzidine (DAB). Before mounting coverslips, glycerol was applied. Cells positive for TUNEL were counted using the formula TUNEL/DAPI × 100%.

### Network pharmacology

#### The acquisition of medicinal components and related targets of resveratrol

The gene targets for resveratrol were identified in the Swiss Target Prediction and PharMapper online databases^14^.

#### SCI target genes

The Treatment Targets Database (TTD) and GeneCards were searched for action targets associated with SCI. All collected CR and SCI targets were merged prior to Uniprot database rectification. Common target genes were identified using Venn diagrams (http://bioinformatics.psb.ugent.be/webtools/Venn/). The experiment adhered to the principles outlined in the Declaration of Helsinki (2013 revision)^15^.

#### Construction of the protein–protein interaction (PPI) network

Targets were inputted into the STRING database to establish relationships in the target interaction network, with a minimum interaction score of 0.4, and isolated points were removed. The Cytoscape3.7.2 software was used to import the PPI data (string_interactions.tsv) and to construct the PPI network.

#### Gene ontology (GO) and KEGG pathway enrichment analysis

Three aspects are considered when analyzing GO functional enrichment for potential targets, namely biological processes (BPs), cellular components (CCs), and molecular functions (MFs). Each category was ranked by significance, and the top 10 enriched items were displayed in bar charts and bubble charts. Pathway diagrams were generated using the 'pathview' package for enriched KEGG pathways with correlative targets^16^.

### Western blot

28 days following SCI, euthanized rats under isoflurane anesthesia. Samples were then collected and fixed in 4% paraformaldehyde. A 0.5 cm long sample tissue was homogenized in 300 μL of lysate-containing protease inhibitors. Following lysate preparation, the electrophoresed tissue lysate was transferred to polyvinylidene difluoride membranes. Next, the membranes were blocked with 5% skimmed milk and incubated in an incubator for 60 min. The membranes were further blocked for two hours and then incubated with primary antibodies, consisting of anti-JNK (1:1000; Proteintech), anti-p-JNK (1:1000; Proteintech), anti-p-p38 (1:500; Bioss), anti-p38 (1:1000; Bioss), and GAPDH (1:2000, UtiBody). They were then incubated with secondary antibodies (1:3000; Bioss) in an incubator for 1 h. ImageJ was used to quantify grey bands.

### Enzyme-linked immunosorbent assay (ELISA) analysis

The expression levels of IL-1β, TNFα, and IL-6 after resveratrol administration were measured using ELISA. ELISA assays were performed using the Rat TNF-α ELISA Kit (bsk13003, BIOSS, Beijing), Rat IL-1β ELISA Kit (bsk13006, BIOSS, Beijing,) and Rat IL-6 ELISA Kit (bsk13004, BIOSS, Beijing) according to the manufacturer's protocols. In each sample, the levels of IL-1β, TNF-α, and IL-6 were expressed as the relative density of the concentrations in the SCI rat group.

### Statistical analysis

Statistical analyses were carried out using GraphPad Prism (GraphPad Prism 8; GraphPad). Data were presented as mean ± SEM. Statistical differences within groups at different time points were analyzed by one-way analysis of variance. The Student's t-test was used for pairwise comparisons. P-values less than 0.05 were considered statistically significant.

## Results

### Resveratrol treatment improves mobility in post-SCI rats

**Figure 1:**
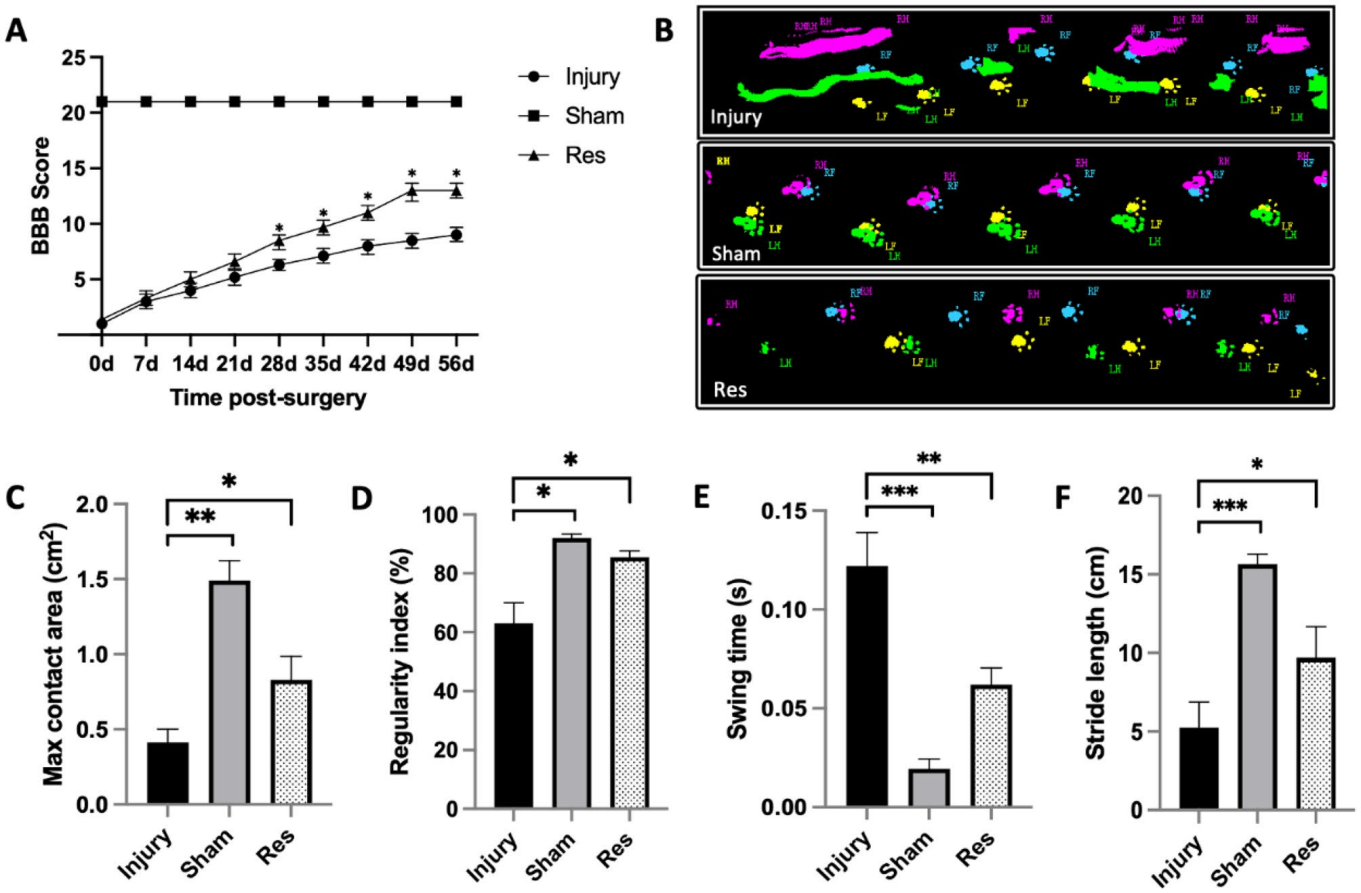
Resveratrol therapy improved motor function of post-SCI rats. (**A**) BBB locomotor score were performed from 0 to 56 days post-injury. (**B**–**F**) Catwalk gait analysis was performed to assess hindlimb motor function objectively at 56 days post-injury, we selected four commonly used indicators include max contact area, regularity index, swing time and stride length. (*p < 0.05, **p < 0.01, ***p < 0.001; *BBB* Basso, Beattie, and Bresnahan, *Res* resveratrol, *SCI* spinal cord injury).

**Figure 2:**
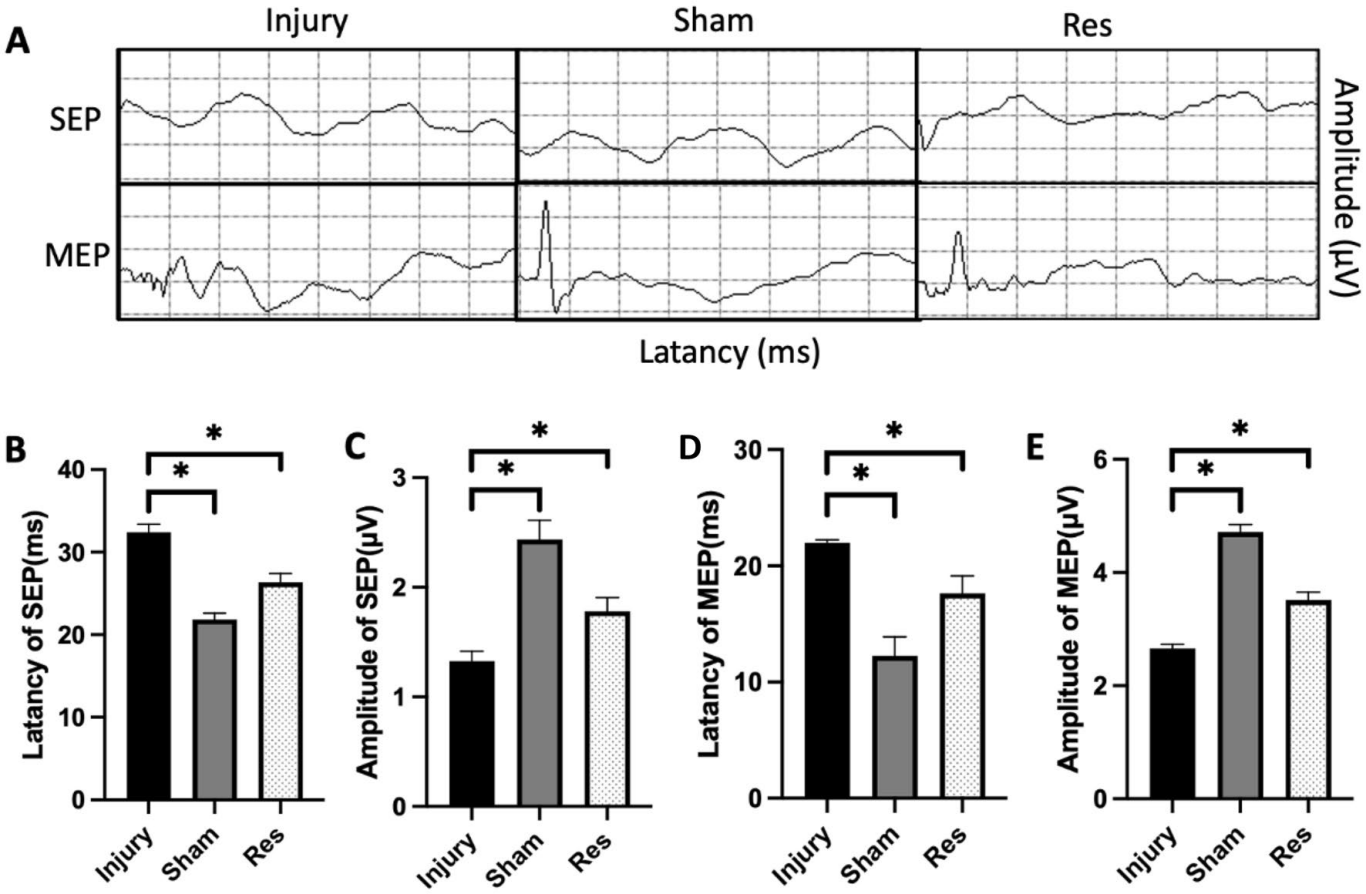
Resveratrol enhanced functional recovery after SCI. (**A**–**E**) Representative image of SEP and MEP (average of 100 responses) from the injury groups, sham groups, and Resveratrol groups (*P < 0.05, **P < 0.01, ***P < 0.001; *SEP* somatosensory evoked potential, *MEP* motor evoked potential).

As previously mentioned, BBB scores were employed to assess the recovery of locomotor performance in rats (Fig. 1A). The BBB score for the injury and resveratrol groups was 21 before spinal cord injury and 1 on the first day after injury. Interestingly, the BBB scores in the injury and resveratrol groups were comparable from day 1 to day 21. However, at 28, 35, 42, and 49 days post-SCI, the BBB score in the resveratrol group was significantly higher than that in the injury group, suggesting that resveratrol can improve motor function after SCI (n = 9, all the data are expressed as means ± SD, analyzed using ANOVA followed by Tukey's post hoc test). The catwalk gait analysis system, as an objective tool, was also used to examine rat mobility 56 days after SCI. Rats in the resveratrol group exhibited a substantial recovery in exercise capacity compared to those in the injury group. Significant differences were also noted in maximum foot contact area, regularity index, swing time, and stride length between the injury and resveratrol groups (Fig. 1B–F). Electrophysiological evaluation also uncovered longer latency and lower amplitude in SEP and MEP in rats of the injury group than those in the sham group (Fig. 2A–E).

After 28 days of resveratrol treatment, the latency of SEP and MEP was shorter in the resveratrol group (Fig. 2B,D); MEP and SEP amplitude was higher than that in rats of the injury group (Fig. 2C,E).

### Resveratrol mitigates spinal cord damage after SCI

**Figure 3:**
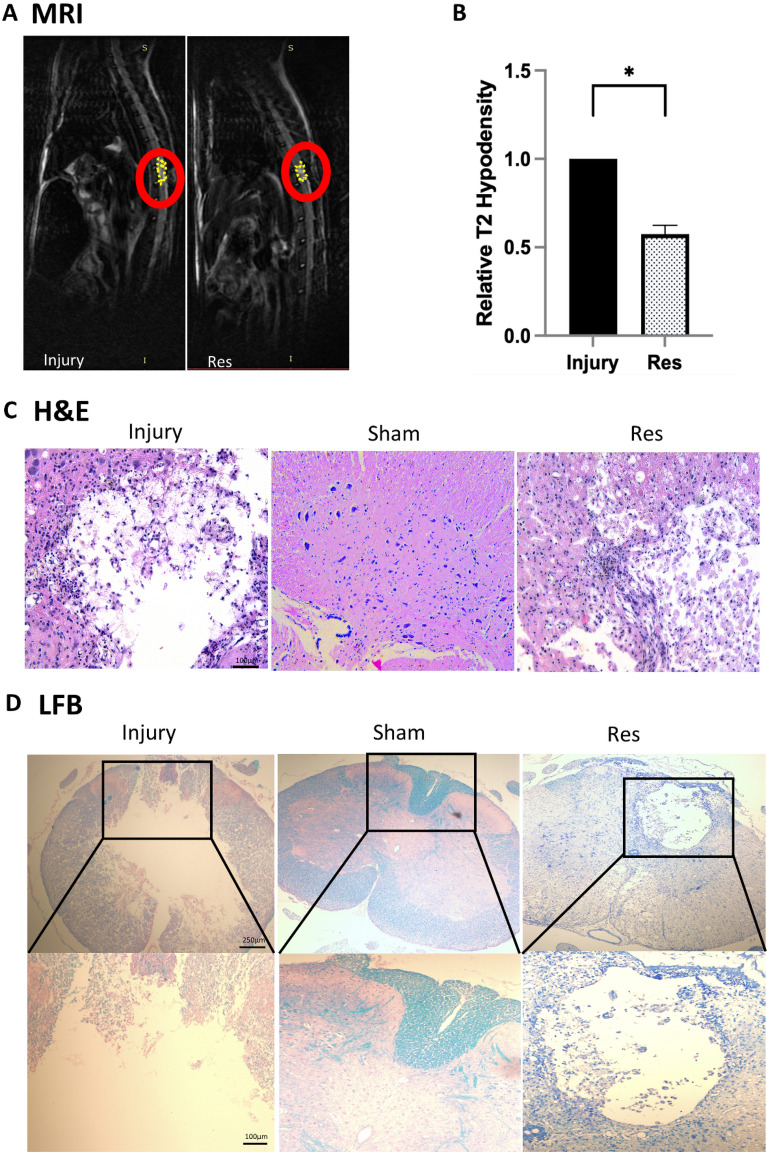
Resveratrol reduced tissue damage after SCI. (**A**,**B**) Representative MRI T2 image of injury and Resveratrol treatment groups. The T2 intensity was semiquantified using ImageJ software as the lesion size. (**C**) Representative H&E staining of spinal cord sections from the injury groups, sham groups, and Resveratrol groups at 4 weeks after surgery. (**D**) Representative micrograph showing myelin sparing at the damaged area in sections stained with LFB at 4 weeks after surgery. (*P < 0.05, **P < 0.01, ***P < 0.001, *H&E* haematoxylin and eosin, *LFB* Luxol fast blue, *MRI* magnetic resonance imaging, *Res* resveratrol, *SCI* spinal cord injury).

To elucidate the correlation between motor function recovery following resveratrol treatment and the amelioration of spinal cord damage, the extent of spinal cord hyper-signal in the resveratrol and injury groups was assessed 4 weeks after SCI using MRI, and the data were quantified. The analysis determined that resveratrol could dramatically reduce the lesion area at the site of injury (Fig. 3A,B).

Next, analysis of HE-stained spinal cord sections 28 days after SCI highlighted that the resveratrol-treated group exhibited a higher tissue density than the injury group (Fig. 3C). Meanwhile, the area of injury was smaller in the resveratrol group compared to that in the injury group. In the fourth week, a portion of rats experienced a deterioration in vital status, and thus, this time point was selected for LFB staining. The outcomes showed increased myelin preservation in the resveratrol group compared to the injury group (Fig. 3D). Consistent with the HE staining results, the area of demyelination was larger in the injury group compared to that in the sham group. Importantly, the area of demyelination was smaller in the resveratrol group compared to that in the injury group. Our findings collectively suggest that resveratrol treatment significantly relieved post-SCI injury, as evidenced by improved myelin preservation and spinal cord tissue density.

### Resveratrol improves neural survival and limits astrocyte proliferation after SCI

**Figure 4:**
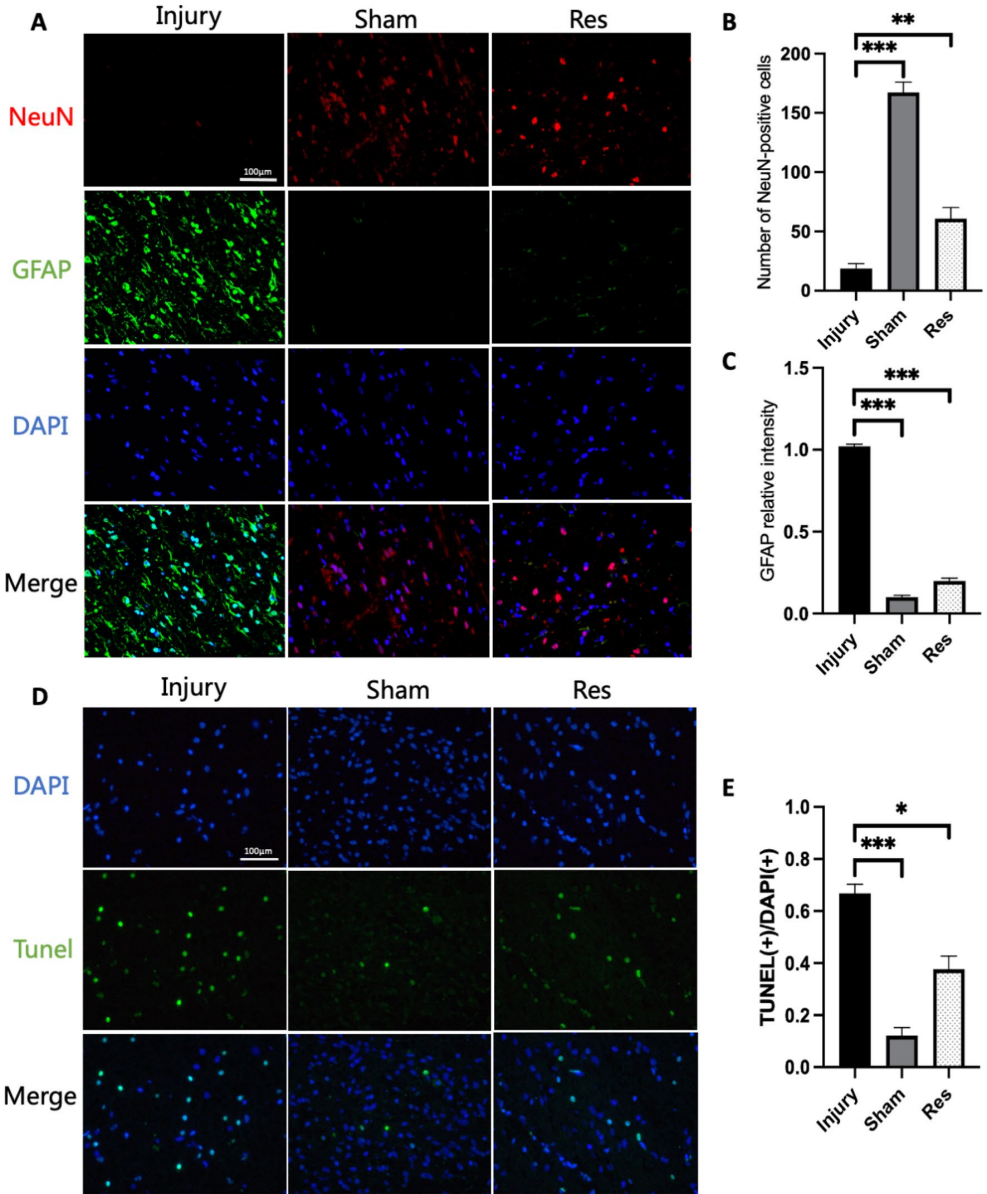
TUNEL staining after spinal cord injury and immunofluorescence staining for NeuN and GFAP in the ventral horn of the injury epicenter. (**A**) Representative photo of NeuN (in red), GFAP (in green) and DAPI (in blue) staining at 4 weeks after SCI in different groups. (**B**) Quantitative analysis of neuronal cells. (**C**) Quantitative analysis of GFAP relative intensity. (**D**) Representative TUNEL-positive cells (in green) in spinal cord sections at 4 weeks after SCI. Nuclei were stained with DAPI (in blue). (**E**) Quantitative analysis of the TUNEL/DAPI (*GFAP* glial fibrillary acidic protein, *DAPI* 4',6-diamidine-2'-phenylindole dihydrochloride, *SCI* spinal cord injury; *P < 0.05, **P < 0.01, ***P < 0.001).

The protective effects of resveratrol on neurons were explored using NeuN staining (Fig. 4A). The number of NeuN + cells was smaller in the injury group compared to that in the sham group. 28 days after SCI, the results showed that the number of NeuN + cells was bigger in the resveratrol group than in the injury group (Fig. 4B). Overall, these results indicate that resveratrol improved neuronal survival in spinal cord tissue after SCI.

In order to examine the influence of resveratrol on neurogliosis, the production rate of GFAP was determined. At 28 days post-SCI, the relative immunofluorescence intensity of GFAP-positive glial cells was higher in the injury group compared to that in the resveratrol group (Fig. 4C).

### Resveratrol decreases apoptosis after injury

TUNEL staining was employed to detect the apoptotic rate 4 weeks after SCI (Fig. 4D). The results showed that the relative fluorescence intensity of TUNEL-positive cells was significantly lower in the sham group compared to that in the injury group. Moreover, the relative fluorescence intensity of TUNEL-positive cells was lower in the resveratrol group compared to that in the injury group (Fig. 4E). Overall, the results established that resveratrol preserved the spinal cord by attenuating apoptosis.

### Resveratrol regulates the activity of the MAPK pathway and inhibits the expression of inflammatory factors after SCI

**Figure 5:**
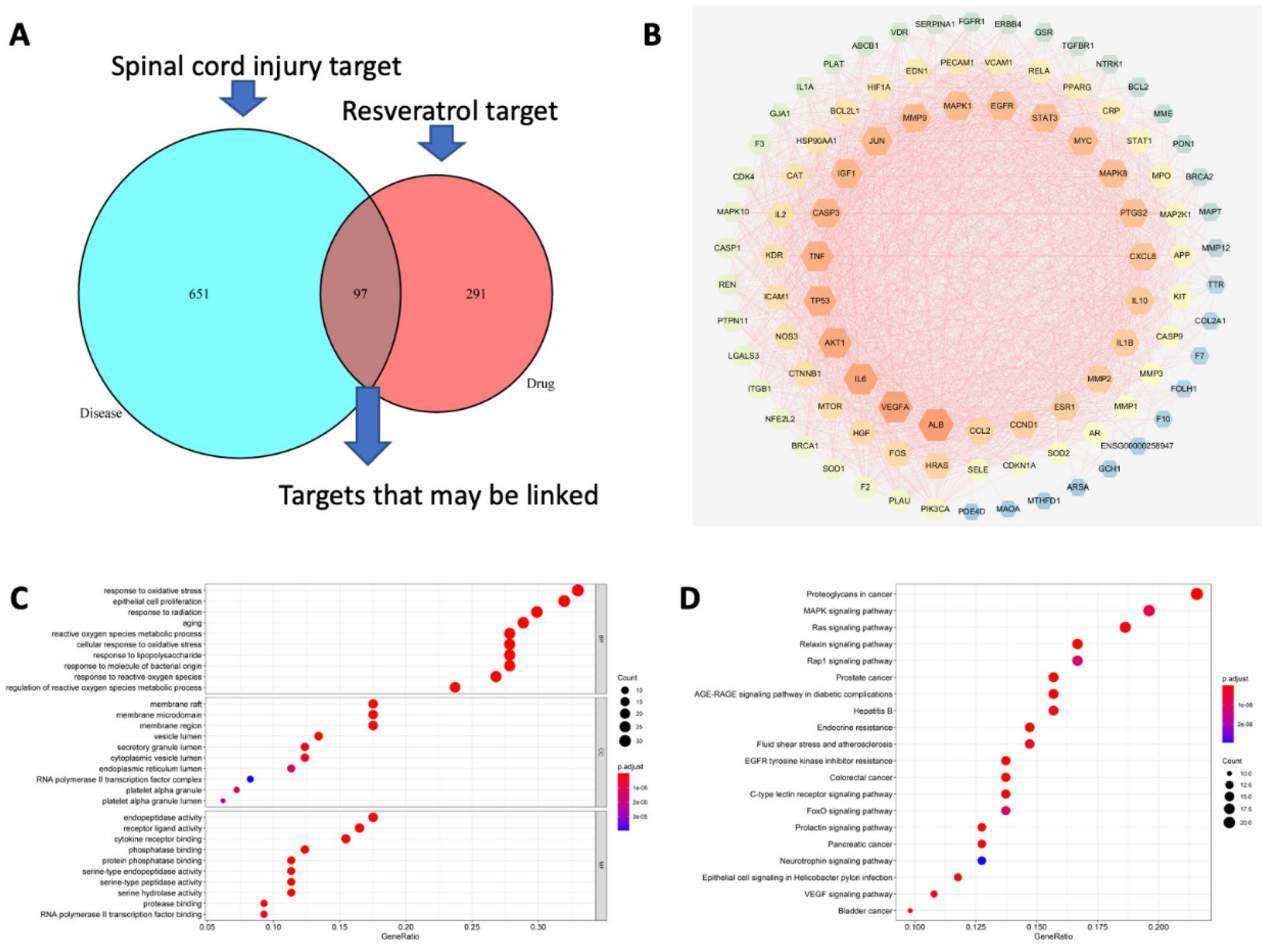
Pathway of resveratrol regulating inflammation (**A**) Common target genes between Resveratrol and SCI were obtained using Venn diagrams (http://bioinformatics.psb.ugent.be/webtools/Venn/). (**B**) Import PPI data (string_interactions.tsv) to build a PPI network using Cytoscape 3.7.2 software (**C**) The biological process results of the GO analysis. (**D**) The KEGG analysis (*GO* gene ontology, *KEGG* Kyoto Encyclopedia of Genes and Genomes).

**Figure 6:**
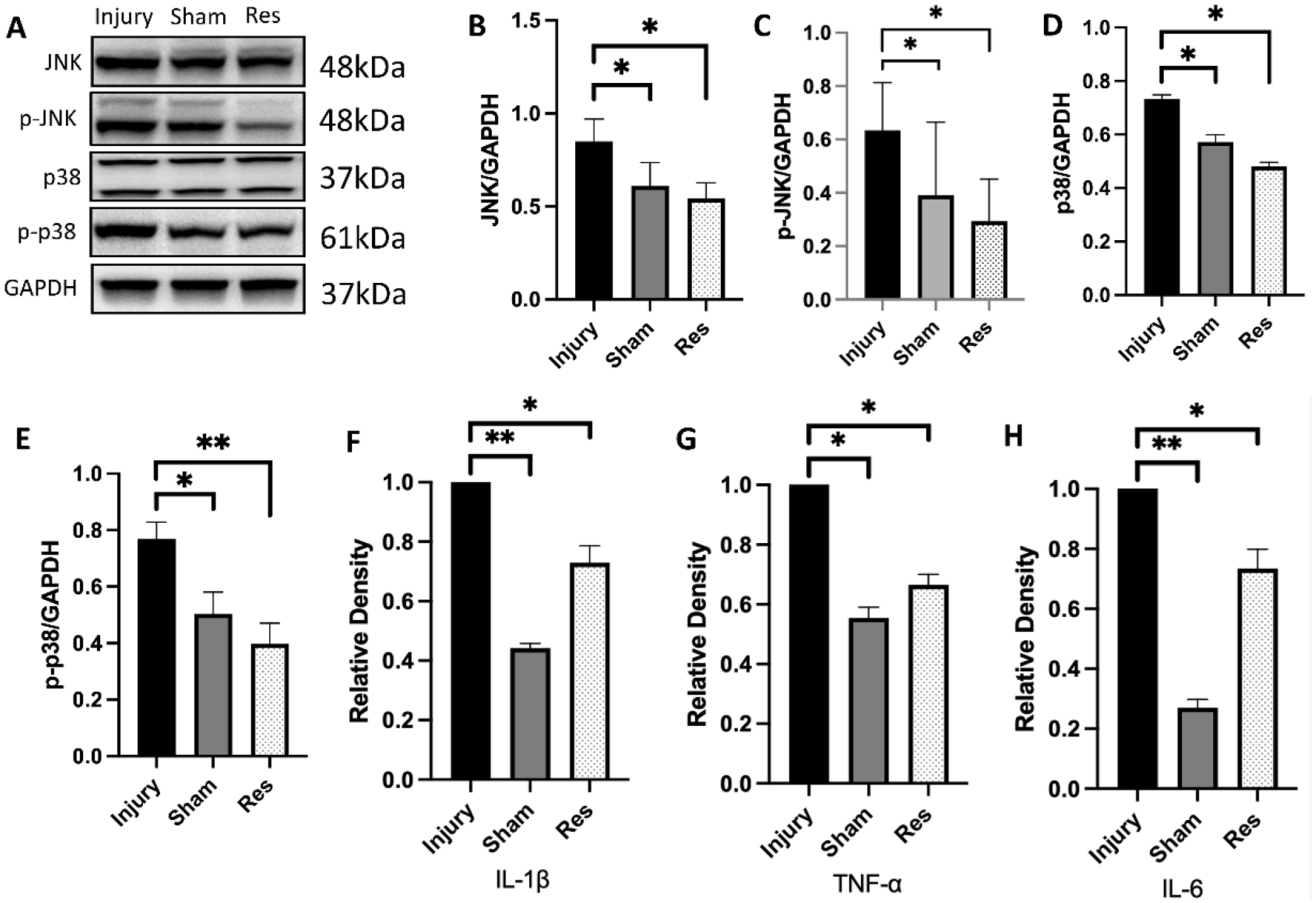
Resveratrol regulates inflammatory proteins and inflammatory factors (**A**) Representative Western blot of p38, p-p38, JNK and p-JNK phosphorylation levels at 4 weeks after surgery. (**B**–**E**) Quantitative analysis of the protein levels (**F**–**H**) Results of inflammatory cytokines levels by ELISA detection between sham group and injury group at 4 weeks post-surgery. (*P < 0.05, **P < 0.01, ***P < 0.001).

The mechanism by which resveratrol exerted these effects was further explored. To begin, GO functional enrichment analysis of potential targets using network pharmacology revealed that resveratrol acted on the MAPK signaling pathway (Fig. 5A–D). Then, the MAPK signaling pathway was modulated to determine the impact of resveratrol on the inflammatory response in the injured region after SCI. Additionally, this study examined the phosphorylation levels of proteins associated with the MAPK signaling pathway in the resveratrol and injury groups at week 4 post-SCI (Fig. 6A). The results showed that the protein levels of JNK, p-JNK/JNK, and p-p38/p38, were lower in the resveratrol group than in the injury group. Likewise, the protein expression levels of p38 were significantly lower in the resveratrol group than in the injury group (Fig. 6B–E). Furthermore, the relative densities of IL-1β, IL-6, and TNF-α were significantly lower in the resveratrol group compared with those in the injury group (Fig. 6F–H). These results conjointly suggested that resveratrol potentially participates in inhibiting the activation of the MAPK signaling pathway and suppresses the release of inflammatory factors post-SCI.

## Discussion

The results of this study showed that resveratrol treatment can promote the recovery of SCI in rat contusion models by inhibiting the activation of the JNK/p38MAPK signaling pathway. In addition, resveratrol treatment suppressed gliosis and increased neuronal survival. Notably, resveratrol could induce the functional recovery of locomotion and decrease demyelination and the size of the lesion. What's more, resveratrol inhibited the release of pro-inflammatory cytokines to protect the tissue from secondary damage resulting from inflammation. The pathological process of SCI is divided into two main parts: mechanical damage resulting from a transient compression to the spinal cord^17–19^, and primary lesions following the injury. Thereafter, a secondary injury cascade can exacerbate neurological dysfunction and lead to permanent damage^20–24^. The inflammatory response promotes apoptosis-mediated neuronal necrosis in the damaged area of the tissue^25^.

As is well documented, inflammation plays a critical role in host defense responses to peripheral inflammation and injury. After SCI, ischemia and disruption of the blood-spinal cord barrier (BSCB) trap the spinal cord in an environment rich in inflammatory cells and cytokines^22,26^. However, inflammatory responses partly contribute to preserving the biochemical properties of the injured region^20^. Spinal cord inflammation increases the permeability of the BSCB owing to the release of large amounts of pro-inflammatory mediators from reactive glial cells, allowing peripheral blood immune cells to infiltrate the area of injury to clear the necrotic tissue^27^. At the same time, glial cells modulate the action of immune cells to protect the tissue from secondary damage caused by excessive inflammation^28^. Therefore, inflammation appears to have a bidirectional effect on SCI. The accumulation of immune cells and the subsequent inflammation further amplify the inflammatory response^29,30^. Herein, the levels of IL-1β, IL-6, and TNF-α were significantly increased in the injury group one week after SCI, which is in agreement with the results of previous studies^31^. The cascade of inflammatory responses accentuates the accumulation of inflammatory metabolites in the injury site, which further increases the permeability of the BSCB^32^. Therefore, our outcomes demonstrated that anti-inflammation appears to be an effective therapeutic method and a crucial basis for neuroprotection after SCI. At the same time, Gene Ontology (GO) analysis determined that the enriched differentially expressed proteins were related to the JNK/p38MAPK signaling pathway. Of note, JNK and p38 MAPK play a key role in the pathogenic mechanism of SCI. Specifically, activation of this pathway contributes to all components of secondary injury, including inflammatory responses, oxidative responses, neuronal death, and glial proliferation. Consequently, targeting the p38/JNK MAPK pathway may be a therapeutic approach for the treatment of SCI.

Prior studies have found resveratrol to exert anti-inflammatory and antioxidant effects^33–36^. At the same time, the cascade effect of secondary injury also up-regulates the expression of pro-inflammatory cytokines mediated by the MAPK signaling pathway, thereby triggering inflammation^37^. Additionally, resveratrol efficiently inhibited the MAPK signaling pathway and its downstream signaling molecules^38,39^. Our results portrayed that the protein and phosphorylation levels of p38, p-p38/p38, JNK, and p-JNK/JNK were higher in the injury group compared with those in the sham group at 28 days post-SCI. Contrastingly, the phosphorylation levels of p38, p-p38/p38, JNK, and p-JNK/JNK were lower in the resveratrol group compared to those in the injury group.

Herein, resveratrol treatment reduced the area of spinal cord contusion and gliosis after SCI and promoted the recovery of motor function, validating its efficacy for the treatment of nerve damage after SCI. In addition, the levels of inflammatory factors were dramatically reduced after resveratrol treatment. Overall, resveratrol treatment enhanced neural survival in rats after SCI. Noteworthily, the role of resveratrol in modulating p38/JNK MAPK may be ascribed to reduced inflammatory cell infiltration and its antioxidant properties. It has been proposed that the protective role of resveratrol against injury progression is closely associated with the functional blockade of the p38/JNK MAPK signaling pathway^5^.

## Conclusion

In short, the administration of resveratrol protected motor function and neuronal survival in rats after SCI. Furthermore, this study corroborated that resveratrol exerts its anti-inflammatory effects by inhibiting the JNK/p38MAPK signaling pathway (Supplementary Information S1).

### Supplementary Information

Supplementary Information.

## Data Availability

All data can be found in the manuscript. The datasets used and analyzed during the current study available from the corresponding author on reasonable request.

## Abbreviations

SCI: Spinal cord injury
CNS: Central nervous system
MAPK: Mitogen-activated protein kinases
TNF-α: Tumor necrosis factor-α
IL-1β: Interleukin-1β
JNK/p38MAPK: C-jun N-terminal kinase/p38 mitogen-activated protein kinase
HE: Haematoxylin and eosin
LFB: Luxol fast blue
GFAP: Glial fibrillary acidic protein
GO: Gene ontology
SEP: Somatosensory evoked potentials
MEP: Motor evoked potentials
PPI: Protein–protein interaction
KEGG: Kyo to encyclopedia of genes and genomes
ELISA: Enzyme-linked immunosorbent assay
BSCB: Blood-spinal cord barrier
