# Author Correction: Resveratrol improves the prognosis of rats after spinal cord injury by inhibiting mitogen-activated protein kinases signaling pathway

**DOI:** 10.1038/s41598-023-49400-x

**Published:** 2023-12-19

**Authors:** Shunli Kan, Chengjiang Liu, Xinyan Zhao, Sa Feng, Haoqiang Zhu, Boyuan Ma, Mengmeng Zhou, Xuanhao Fu, Wei Hu, Rusen Zhu

**Affiliations:** Department of Spine Surgery, Tianjin Union Medical Center, 190 Jieyuan Road, Hongqiao District, Tianjin, 300121 China

Correction to: *Scientific Reports* 10.1038/s41598-023-46541-x, published online 13 November 2023

The Funding section in the original version of this Article was incomplete.

“This work was funded by Tianjin Union Medical Center (2022JZXK05, 2022JZXK02, 2023YJZD002), Tianjin Key Medical Discipline (Specialty) Construction Project (TJYXZDXK-064B).”

now reads:

“This work was funded by Tianjin Union Medical Center (2018YJ010, 2022JZXK05, 2022JZXK02, 2023YJZD002), Tianjin Key Medical Discipline (Specialty) Construction Project (TJYXZDXK-064B).”

The original Article has been corrected.

