# Supplementary figures and images for: Resveratrol improves the prognosis of rats after spinal cord injury by inhibiting mitogen-activated protein kinases signaling pathway
Source: Sci Rep. 2023 Nov 13;13:19723. doi: 10.1038/s41598-023-46541-x (PMC10643657; doi:10.1038/s41598-023-46541-x)

JNK

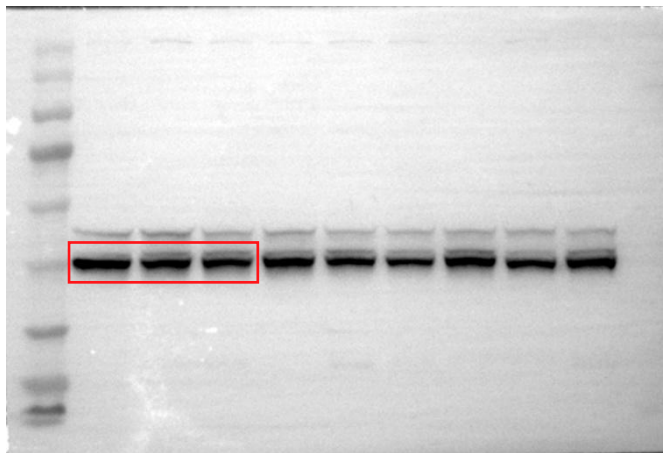

Marker

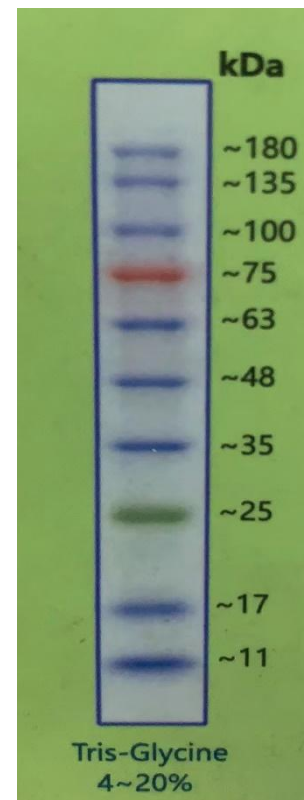

GAPDH

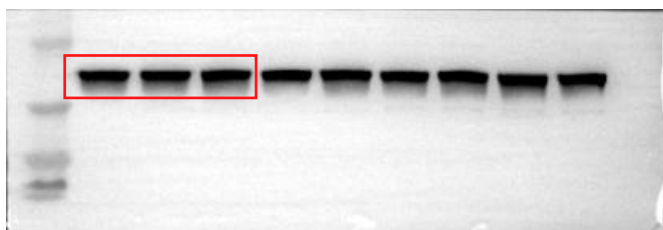

Marker

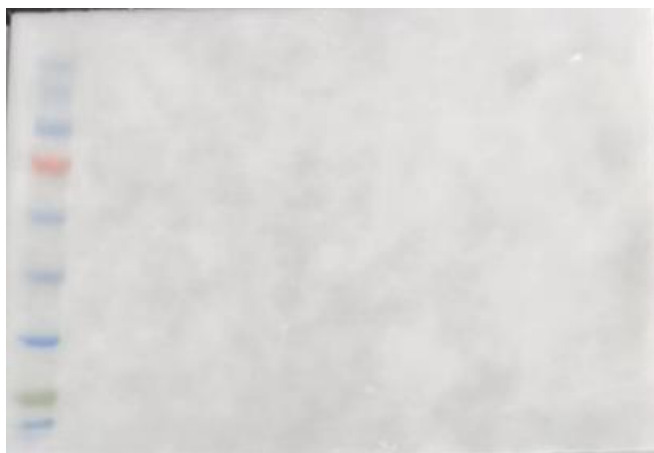

p-JNK

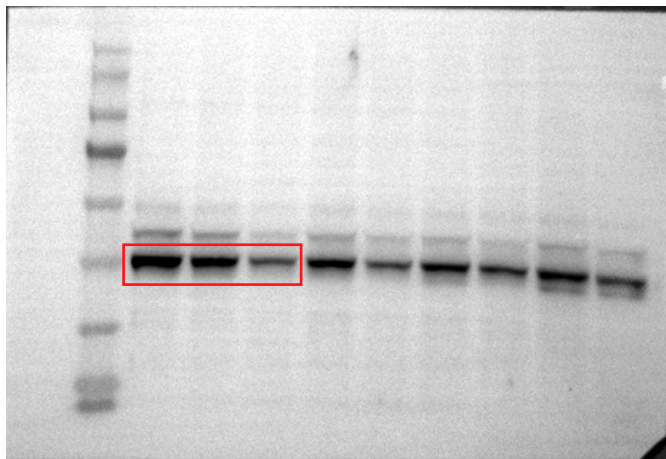

GAPDH

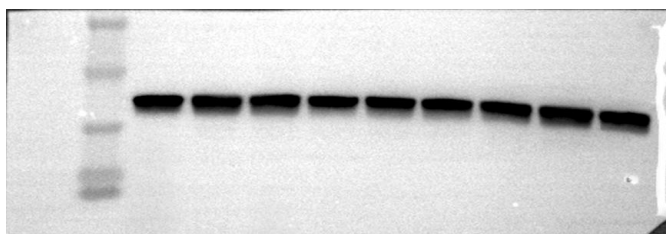

Marker

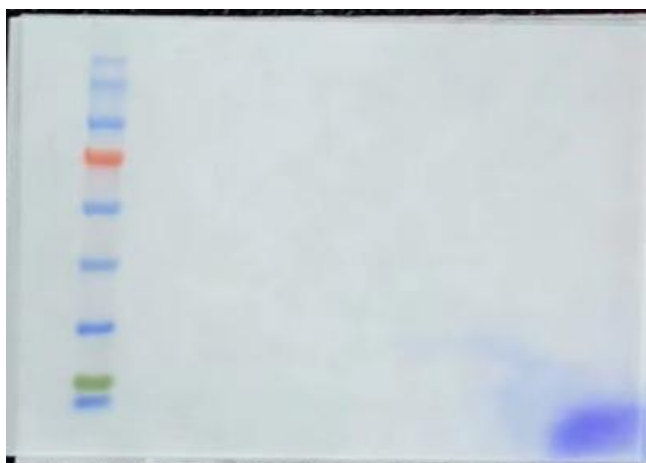

p38

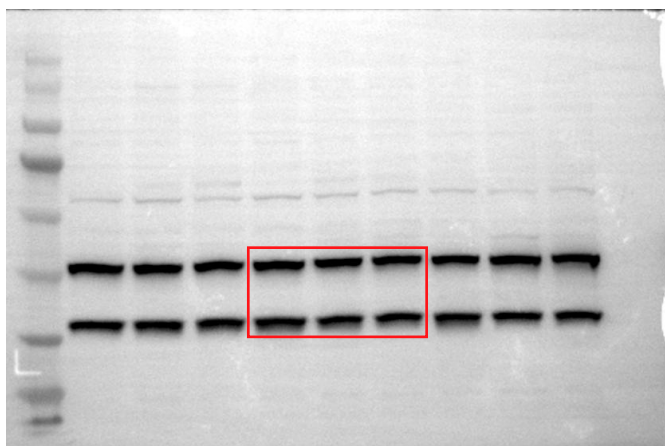

GAPDH

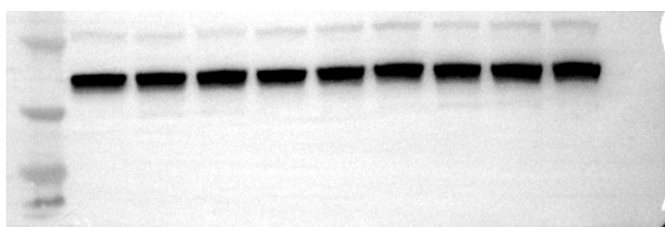

Marker

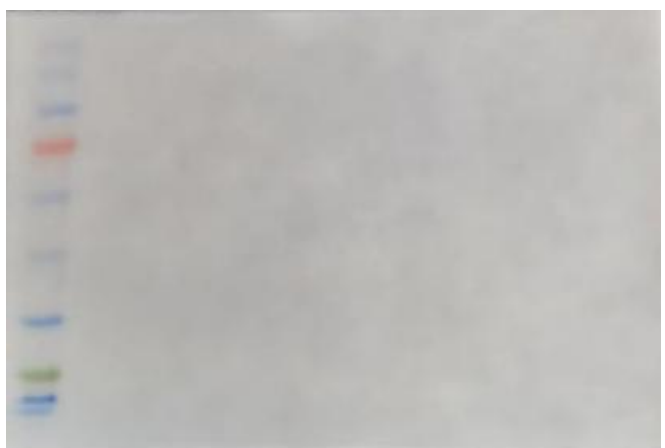

p-p38

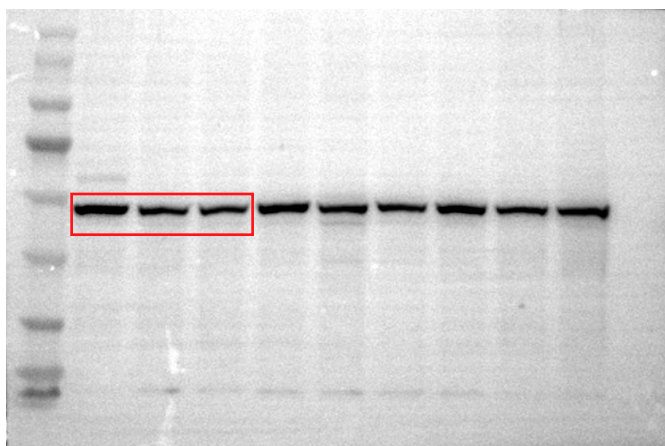

GAPDH

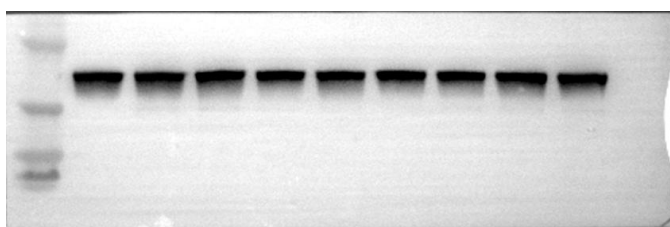

Marker

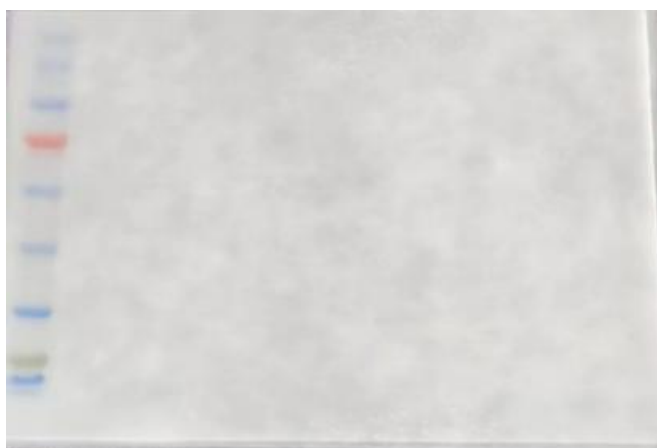

Supplement: Supplementary file 1 — Supplementary Information. [file 41598_2023_46541_MOESM1_ESM.pdf]
